# 

**Published:** 1907-07

**Authors:** 


### BOOKS RECEIVED.

Laboratory Manual of Invertebrate Zoology. By Gilman A. Drew, Ph.D., Professor of Biology at the University of Maine. 12 mo, pp. 201. Philadelphia and London: W. B. Saunders Company. 1907. (Price, $1.25.)

---

Clinical Lectures on Neurasthenia. By Thomas D. Savill, M.D., Lond., physician to the West-End Hospital for Diseases of the Nervous System, London. Small 8 vo., pp. 216. New York: William Wood and Company, 1907. (Price, $1.50.)

---

Nephritis. A Manual of Bright's Disease and of Allied Disorders of the Kidneys, by Seelye W. Little, M.D. 12 mo., pp. 134. New York: The Grafton Press. 1907. (Price, $1.25.)

---

Report of the Commissioner of Education for the year ending June 30, 1905. Vol. 1. Washington: Government Printing House. 1907.

---

The Standard Family Physician, by Dr. Carl Reissig, of Hamburg, Germany, Dr. Smith Ely Jelliffe and others; 2 volumes; large 8 vo.; illustrated. New York and London: Funk & Wagnalls Company. 1907. (Buckram binding, $15.00 per set, three quarters morocco, $20.00, net.)

---

Progressive Medicine, Vol. 1, No. 2, June, 1907. A Quarterly Digest of Advances, Discoveries and Improvements in the Medical and Surgical Sciences. Edited by Hobart Armory Hare, M.D., Professor of Therapeutics and Materia Medica in the Jefferson Medical College of Philadelphia. Octavo, 381 pages, with illustrations. Lea Brothers & Co., Philadelphia and New York. (Per annum, in four cloth-bound volumes, $9.00; in paper binding, $6.00, carriage paid to any address).

---

Gynecology and Abdominal Surgery. Edited by Howard A. Kelly, M.D., Professor of Gynecologic Surgery at the Johns Hopkins University, and Charles P. Noble, M.D., Clinical Professor of Gynecology at the Woman's Medical College, Philadelphia. Imperial octavo, vol. 1, pp. 851. Illustrated by Hermann Becker and others. Philadelphia and London: W. B. Saunders Company. 1907. (Price, $8.)

---

American Practice of Surgery. A Complete System of the Science and Art of Surgery by representative surgeons of the United States and Canada. Edited by Joseph D. Bryant, M.D., and Albert H. Buck, M.D., New York. Complete in eight volumes. Vol. III. Imperial octavo, pp. 775, illustrated. New York: William Wood & Company. 1907. (Price, cloth, $7.00).

---

The Principles and Practice of Dermatology, by William Allen Pusey, A.M., M.D.; Professor of Dermatology in the University of Illinois. Octavo, pp. 1021. Illustrated. New York and London: D. Appleton & Company. 1907. (Price, $6.00.)

---

Manual of Disease of the Nose, Throat, and Ear. By E. B. Gleason, M.D.; Clinical Professor of Otology in the Medico-Chirurgical College of Philadelphia. 12 mo. pp, 556. Illustrated. Philadelphia and London: W. B. Saunders Company. 1907. (Price, flexible leather, $2.50 net.)

The Practical Medicine Series of Year Books. Ten volumes. Issued under the general editorial charge of Gustavus P. Head, M.D., Professor of Laryngology and Rhinology in the Chicago Post-Graduate Medical School. Vols. 1, 2, and 3, General Medicine, Surgery, Eye, Ear, Nose, and Throat. Series 1907. Chicago: The Year Book Publishers. (Prices, $1.25, $1.50, $2.00; entire series, $10.00).

### LITERARY NOTE.

### ITEMS.

### INDEX TO VOL. LXII.

### AUGUST, 1906—JULY, 1907.

PAGE

ABORTION, criminal, and illegitimacy .......416
following use of diachylon .......420
Accidents, report of required.....621
Achylia gastrica .................655
Address to graduates ............ 1
Adrenalin solutions in hay fever.. 55
Air we breathe...................339
Amblyopia, monocular hysterical.673
American Association for Advancement of science...295
medicine, leading men and events in history of. 40
Medical Association officers ..................755
Amputations, concerning ........730
Andrews, Champe S., safeguards to protect people from quack doctors ......................... 98
Anemia and incipient tuberculosis, treatment by hypodermic medication .........................537
Anesthesia, eucain in rectal diseases ..........................551
Anesthetics, local, in operations on chest and abdomen .........131
some new local...... 83
Antisclerosin and blood salts.....624
Appendicitis, observations on 500 operations for .....146
plea for earlier diagnosis of ...........384
Arteriosclerosis, treatment of.....480
Articulations, the pelvic.......... 93
Attack on physician, unfounded..622
Automobiles in New York.......698

BEAHAN, A. L., plea for earlier diagnosis of appendicitis......384
Bedouins in Africa...............698
Bier's passive congestion treatment ......................... 91
Bile duct, obstruction of the common ............................228
Birchmore, Woodbridge Hall, end of the season illness............201

PAGE

Birchmore, Woodbridge Hall, logic of the situation...........455
Blenorrhea, treatment of in women ........................548
Blindness, prevention of unnecessary......................491, 493
Blood salts, administration of.....736

**Books and Authors:**
Ashton: Textbook of gynecology ................125, 639
Attfield: Manual of chemistry.578
Austin: Clinical chemistry....642
Bacon: Manual of otology...512
Baile: Textbook of histology.513
Bailey: Diseases of nervous system from traumatism...244
Bainbridge: Operative gynecology ..................310
Bernays: Golden rules of surgery ......................310
Billings: Modern clinical medicine, vol. 3.............370
Bishop: Ear and its diseases..574
Blakiston: Physicians' visiting list, 1907...............303
Bovee: Practice of gynecology ......................241
Brickner: Surgical suggestions .......................306
Brown: Eczema..............310
Bryant: American practice of surgery, Vol. 1, 301; vol. 2,.568
Bulkley: Diseases of skin and internal disorders ..........121
Bulkley: Menstrual function and diseases of skin........ 66
Burr: Primer of psychology and mental disease.........579
Butler: Materia medica, therapeutics, etc ...............244
Cabot: Case teaching in medicine .......................305
Carr: Pediatrics..............369
Chance: Treatment of bodily deformities ................ 66
Coleman: Syllabus of materia medica ...................644
DeLee: Obstetrics for nurses.440

PAGE

Directory of New York, New Jersey and Connecticut, 1906 ......................... 639
Dorland: American illustrated dictionary ................575
Einhorn: Diseases of the stomach ...................436
Ellis: Demonstrations of anatomy ....................123
Ellis: Psychology of sex, vol. 5 .........................639
Emerson: Clinical diagnosis..437
Fischer: Care of baby.......306
Forchheimer: Treatment of internal diseases............366
Fowler: Operating room and patient ....................125
Fowler: Treatise on surgery....179
Friedenwald: Diet in health and disease................573
Fruhwald: Diseases of children .........................242
Gould: Biographic clinics, vols. 4 & 5................708
Gould: Operations on stomach and intestines.............638
Gould: Practitioner's medical dictionary .................641
Grayson: Diseases of nose, throat and ear.............513
Hampton-Robb: Nursing ....120
Hare: Progressive medicine, vol. 8, no. 2, 246; no. 3, 303; no. 4, 569; 1907, vol. 1,.....708
Harvey lectures..............510
Head: Practical Medicine Yearbooks, 1906, vol. 1, general medicine, 243; vol. 2, general surgery, 243; vol. 3, eye, ear, etc., 307; vols. 4-5, gynecology, obstetrics, 372; vol. 8, materia medica, etc., 576; vol. 7, pediatrics and orthopedic surgery, 577; vol. 6, general medicine, 578; vol. 9, anatomy, physiology,.579
Hertzmann: Urinary analysis, etc. ......................119
Hirsch: Genitourinary diseases and syphilis.........373
Hirst: Textbook of obstetrics.640
Hill: Normal histology......643
Howe: Muscles of the eye, vol. 1......................564
Jennings: Hygiene of pregnancy ......................763
Keen: Surgery, vol. 1,........365
Kelly: Walter Reed and yellow fever...................304
Kemper: World's anatomists..247
Kilmer: Physical examination of children ...............126

PAGE

Koplik: Diseases of infancy and childhood..............123
Landis: Compend of obstetrics .......................309
Lea: Practitioners' visiting list, 1907 ..................371
Leffman: Medical chemistry..127
Lippincott: International Clinics, 16th series, vol. 2, 305; vol. 3, 368; vol. 4, 571; 17th series, vol. 1,..............707
Lusk: Science of nutrition....643
Manton: Human embryology.578
Marks: Artificial limbs.......641
Massey: Gynecology and therapeutics ...............706
McConnell: Manual of pathology ................642
Mendel: Textbook of psychiatry .......................635
Morrow: Care of injured.....640
Morris: Materia medica, etc..126
Morton: Genitourinary diseases aud syphilis..........434
Moynihan: Abdominal operations .......................574
Northcote: Christianity and sex problem................176
Osborne: Materia medica and pharmacology .............307
Parke: Human sexuality.....303
Pattee: Practical dietetics ...577
Paul: Nursing in acute infectious fevers.............247
Pedersen: Medical Epitome Series; Materia medica and therapeutics (Kiepe) 373; Pathology (Stenhouse).....575
Peterson: Practice of obstetrics .......................705
Potter: Materia medica and therapeutics ...............311
Powell: Pocket medical formulary .......................644
Prieswerk: Atlas of dentistry.642

**Reports:**

Commissioner of Education, 1904 ......................637
Department of health, Buffalo, 1905..................373
Henry Phipps institute, 1905..309
State board of health, Michigan, 1904.................303
State commission in lunacy, N. Y., 1904................179
Surgeon-general public health and marine hospital service, 1905 ......................309

Page
Reed: Diseases of stomach and intestines ..............511
Rockwood: Manual of physiological chemistry.......... 67
Sachs: Nervous diseases of children ....................122
Schmidt: Test diet and function of the intestines ......245
deSchweinitz: Diseases of the eye ......................245
Shoemaker: Materia medica and therapeutics ..........576
Sobotta: Atlas of human anatomy, vol. 2.................762
Sobotta: Atlas and textbook of anatomy, vol. 1,.........439
Sollmann: Textbook of pharmacology .................642
Squibb: Materia medica......308
Starr: Nervous diseases......636
Stengel: Textbook of pathology .....................642
Stevenson: Photoscopy ......438
Stevens: Motor apparatus of the eyes..................371
Stoney: Materia medica for nurses .....................643
Theobold: Prevalent diseases of the eye.................435
Thorington: The ophthalmoscope ....................124
Thorington: Retinoscopy.....643
Thornton: Dose book and prescription writing........440
Thornton: Pocket medical formulary .................372
Thornton: Pocket medical formulary .................641
Tigerstedt: Textbook of physiology ....................572

**Transactions:**

American Gynecological Association, 1905.............246
American Laryngological Association. 1905, 127; 1906,...644
American Medico-Psychological Association, 1905......304
American Roentgen Ray Society, 1905.................305
American Surgical Association, 1905, 67; 1906,.........638
Medical Association State of Ala .......................308
Section on Obstetrics and Diseases of Women, A.M.A., 1905, 308; 1906,............579
Southern Surgical and Gynecological Association, 1905.306

Page
Treat: International medical annual, 1906................124
Tuttle: Diseases of children..513
Walker: x-rays in general practice ..................307
Ware: Plaster of paris.......512
Wells: Psychology ..........709
Whitman: Orthopedic surgery ......................... 570
Wilcox: Genitourinary and venereal diseases...........122
Witthaus-Becker: Medical jurisprudence, vol. 1,.......367
Witthaus: Manual of chemistry .....................511
Wolff: Practical dermatology 441
Wood: Medical Record visiting list, 1907...............302
Books Received 127, 180, 247, 311, ......374, 441, 514, 580, 644, 709, 763

Boswell, Charles O., leukocytosis in surgical infections...........523
Bowel, obstruction of............647
Brown, C. W. M., infantile scurvy 377
William M., version or high forceps, which and when? ................529
Brush, Edward N., address to graduates ...................... 1
Buffalo Medical Journal, new offices of........................427

CALOX, a preservative of the teeth ........................233
Cannaday, John E., iodine in surgical work ....................669
Carcinoma cutis ................672
Carpets, Oriental, contagion from.359
Carroll, Lieutenant James, promotion of ....................505
Catgut, collargolised ............482
Cerebral abscess, case of.........389
Cesarean operation under difficulties ...................726
section performed by cow .................419
Cholelithiasis, probilin treatment of ............................ 94
Chorea .......................282
Circulation, story of discovery of. 20
Clinton, Marshall, local anesthetics in operations on chest and abdomen.131
obstruction of the bowel....647
Collargol in various affections....736
Collargolum in puerperal fever..224
in surgery ..........481

PAGE

**College and Hospital Notes:**

Buffalo Children's Hospital ........................433, 564
Buffalo General Hospital ....176
Buffalo General Hospital Association ..................364
Buffalo General Hospital Training School for Nurses.761
Buffalo Homeopathic Hospital ......................300
Buffalo Woman's Hospital Training School for Nurses.176
Emergency Hospital Actors' Fair ......................702
Erie County Maternity Hospital ......................509
Erie County Tuberculosis Hospital ..................508
Fordham Hospital ..........702
Frontier Hospital, Buffalo....300
German Hospital, Buffalo.....363
Jackson Health Resort ......702
King Edward VII Sanatorium for consumptives ......... 65
Lackawanna Steel Company's Dispensary ...............433
Marine Hospital, Buffalo..365,432
McGill University...........635
Nurses' Training School, W. C. A. Hospital, Jamestown..761
N. Y. Post-Graduate Medical School and Hospital.....65,433
N. Y. Skin and Cancer Hospital ..................300,509
Riverside Accident Hospital Dispensary ...........433, 564
Syracuse University .........702
Texas Sanitarium for Tuberculosis ...................635
University of Buffalo Medical Department ..118, 509, 510, 703
United States Military Hospital .....................119
United States Military Hospital at the Presidio.......240
Woman's Hospital, N.Y......364

Colles's fracture, old............692
Conservatism in therapeutics ....661

**Correspondence:**

Appendicitis in European centers—Eugene A. Smith.... 96
Blindness and legislation—F. Park Lewis ...............289
Drevet Manufacturing Company vs. Liquozone Company—Charles Marchand...495
Food preservatives and appendicitis—H. H. Langdon.226
Interstate reciprocity in medical licensure—Samuel G. Dixon .....................290
Medical Department, University of Pennsylvania, raises requirements for admission —Charles H. Frazier ......496
National department of health J. Pease Norton ...........291
Raybrook Hospital for Incipient Consumptives—M. P. Burnham ...................582
Requirements for admission to Yale medical school— Herbert E. Smith .........353
Unnecessary operations on women—Arthur Dean Bevan ....................283
Unnecessary operations on women, a reply—Lewis S. McMurtry .................285

Cures, promising impossible .....621
Curettage: indications, technic and complications .................542
Cystoscope, diagnostic value of...278

DEATH, sudden after labor.....417
Deaver, John B., acute and chronic indigestion ............213
Diabetes mellitus ...............461
"Duhrssen Schnitt" in treatment of puerperal convulsions.......142

EARACHE ...................338
Eastman, Thomas B., observations on 500 operations for appendicitis ....................146
Eclampsia, etiology of...........420
Embryotomy on living infant....276
End of the season illness ........201
Epiphyseal separation of ends of humerus .....................501
Erie county jail and penitentiary, inspection of by President Wickser ...........................358
Expert evidence ..................683
evidence, medical...........680
solution of medical.........681
trials of medical............679
medical, discredited.........681
medical testimony......684, 686
medical witness in court....682
witnesses and fees..........683
Experts, non-partisan ............685
boards of..............686
Eyestrain and crime .............144

FINGER NAIL, growth of......426
Foods and medicines, deceptive guarantee of...............629
Fractures, treatment of...........692

PAGE
Fronczak, Francis E., visions of Mary Czajka ................470

GANGRENE following contact with live wire................330
Gardner, James A., why not? a plea ..........................335
Gastric ulcer, surgical aspect of...445
Gestation, ectopic ...............417
unusual case of.419
Glandular fever ..................156
Glycerophosphates, therapeutics of ..........................734
Goldberg, Sigmund, why minor gynecological operations fail...268
Gonorrhea, arhovin in............549
colloid silver in acute.551
treatment of..........160
treatment of masculine ..................735
Gould, George M., eyestrain and crime .........................144
Greene, Dewitt C., the air we breathe ........................339
Gregg, Milton E., conservatism in therapeutics ...................661
Gynecological operations, why failure of ......................268

HAYD, HERMAN E., "Duhrssen Schnitt" in treatment of puerperal convulsions .........142
Hayd, H. E., suit vs. White Sewing Machine Company 754
Herman E., traumatism of the intestines ............212
Health department, proposed new regulations for ................444
Hearing, preservation of .........479
Hemorrhage, accidental in obstetrics ..........................257
Hemorrhoids, treatment of.......480
Hepatitis, treatment of alcoholic..481
Hill, Henry W., appropriations for Buffalo .......................755
Hymen, labor obstructed by unruptured ..................276, 418
Hypothetical question, the.......687

ILLUSTRATIONS:
Bryant, Joseph Decatur, Portrait ...................... 59
Campbell, William Robertson, portrait ....................759
Jacobson, gangrene after contact with live wire:
Fig. 1, showing left upper extremity ..........331
Fig. 2, showing anterior destruction of left leg.332
Fig. 3, showing lateral destruction of left leg.333

PAGE
Fig. 4, showing gangrenous condition of left leg and foot ...........334
Mayo, William J., portrait.... 58
Rose-Cook, cerebral abscess..392
Wallace, Intestinal resection and anastomosis:
Fig. 1, gut at point of obstruction ...........518
Fig. 2, tube for drainage...519
Fig. 3, thick mesentery....520
Fig. 4, gut resected........520
Figs. 5, 6, 7 & 8, lateral anastomosis with Murphy button.......521, 522
Williams, large pus kidney:
Fig. 1, front view .........153
Fig. 2, section view........153
Wright, treatment of hemorrhage:
Fig. 1, post-placental clot ..262
Fig. 2, placenta after delivery ..............263

Impotence caused by bromo seltzer ...........................267
Indigestion, acute and chronic....213
Insane, criminal responsibility of.618
Instruments, perils of intrauterine.275
Intestinal resection and anastomosis ..........................517
Intestines, traumatism of ........212
Iodine in surgical work..........669

**Items:**
Antikamnia Calendar, 1907...441
Antikamnia Chemical Company, file guaranty.........376
Apollinaris, value of .........376
Apollinaris water .......248, 714
Army examinations ..........516
Battle & Company, dislocations ......................645
Battle & Company, intestinal parasites...............582
Bowery Mission Bread Line..375
Brietenbach Company, M. J., Physician's Memorandum Book, 1907... .............376
Denver Chemical Manufacturing Company, "Antiphlogistine Girl" .................376
Knopf, S. A., prize essay .....764
Kress and Owen Company visited by fire ............582
Moet & Chandon white seal champagne .......248, 376, 714
New York Pharmaceutical Company, "Beauty as a Factor in Disease" ........375
Occupation dusts ............646
Od Chemical Company.......714

PAGE
Public Health and Marine Hospital Service examinations ....................516
State civil service commission examinations ..............764
Tri-state Chess Association..248
U. S. Civil Service examinations ....................645
Vital statistics, legislation for.646

JACOBSON, NATHAN, gangrene following contact with live wire ......................330
Jameson, Thomas, obstruction in region of pylorus..............465
Jenkins, J. F. T., treatment of diseases of respiratory tract ......345
Johnson, William D., case of pancreatic cyst.....................665

KIDNEY, case of large pus.....152
King, James E., student ideals.183
Krauss, William C., need of a trained microscopist ...........716

LABOR, injuries to child's head during .......................420
Labor, painless ..................416

**Leading Articles:**
American Association of Obstetricians and Gynecologists 173 ..................233
Annual report of state cancer laboratory .................424
Army medical corps..........229
Association of Military Surgeons .....................167
As to drinking water ........628
Chicago conference on medical education ..............693
Electric instrument shop.....231
Farce comedy and expert evidence ....................499
Fifty-seventh annual meeting of the A.M.A. ............. 56
Gratwick research laboratory.114
Health department problem..425
Improved municipal sanitation ......................355
Insurance lemons for physicians ....................502
Interstate reciprocity in medical licensure ...............111
Military surgeon, the.........501
Osteopathic vertebra put into place ....................357
Prevention of unnecessary blindness ..................498
Summer beverages ..........696
Tearing aside the veil........504
Thaw case, the..............626

PAGE
Typhoid fever and filtration..554
Unnecessary operations on women ..................293
University, a new............627
University of Buffalo ........752
Water supply of Buffalo......422

Leukocytosis in surgical infections ...........................523
Levy, I. H., achylia gastrica......655
Lewis, Denslow, the social evil..249
Library, new, college of Physicians, Philadelphia.............360
Life, warning against the strenuous ...........................233

**Literary Notes:**
Alexander, Practical history state of N. Y...............129
Annals of Otology, Rhinology and Laryngology ..........516
Annals of Surgery ...........712
Annals of Surgery, July number .........................764
Blakiston's Son & Co., Diseases of children........... 68
Blakiston's Son & Co., Foods and their adulteration—Wiley ....................443
British Gynecological Journal.713
Brooklyn Medical Journal....375
Folia Therapeutica...........712
Folia Urologica..............581
Gazette Medical de Paris.....375
Index Medicus ..............711
International Journal of Therapy ...................181
International Medical Review.181
Journal of Ophthalmology and Oto-Laryngology ..........712
Knopf, Dr. S. A., prize monograph ....................713
Leonard's Illustrated Journal.712
Long Island Medical Journal.374
Mellin's Food Co., Diet after weaning .................. 68
Mississippi Valley Medical Association, prize essay......516
Oklahoma Medical News Journal ....................375
Quarterly Journal of Inebriety ...............130, 582
Saunders, W. B. & Co., Announcement of books......129
Saunders, W. B. Company, Illustrated catalogue.......
St. Louis Medical and Surgical Journal and Medical Mirror combined .................314
Therapeutic Gazette, Medical Age and Medicine consolidated ....................314

PAGE
Logic of the situation............455
Low, Charles E., physician-civilian.723
Frank W., prevention of tooth decay ..............388
Lucid, M. M., epiphyseal separation of ends of humerus........501
Lunatic, a responsible............622

McKEE E. S., treatment of sciatica .....................733
McKelway, St. Clair, chancellor's address ........................279
McMurtry, L. S., surgical and serum treatment of puerperal sepsis .........................473
Macpherson, J. D., nature, the good physician ................462
Malpractice suit ended ..........621
Medical bill, new ................697
inspection of schools.....505
Mirror discontinued......630
officer of state forces, training of ..............191
Melena neonatrum ..............155
Microscopist, need of trained .....716
Miller, John, curettage: indications, technic and complications.542

**Miscellany:**

Association of Military Surgeons, U.S. ................130
Buffalo Health Department circulars ..................182
Morse International Agency, New York and London .... 68
Public Health and Marine Hospital Service examinations ...................... 68
State Civil Service examinations .........130, 314, 444, 713

Morris, Robert T., heteroplastic ovarian grafting ...............393
Morse, John Lovett, diseases of nasopharynx in infancy .......598

NASOPHARYNX, diseases of in infancy.598
Nasopharynx, treatment of chronic infections of................. 80
Nature, beautiful law of..........426
the good physician.......462
Nervous diseases, etiology of functional ..................... 71
Nobel prize, awards of...........360
Nurse, the French trained........232

OBITUARY:
Baethig, Henry............... 6[illegible]
Bergmann, v. Ernst..........557
Bidaman, Weston D.........506
Bontecou, Reed Brockway....632
Buhl, Theodore H...........631
Calabrese, Bernardino........117
Carpenter, H. W ............700
Campbell, William R 630.....757
Colegrove, Clinton...........297
Cornwell, L. W..............298
Dunn, Andrew Thomas.......700
Fowler, George Bingham.....507
Garcelon, Alonzo.............362
Henrotin, Fernand ...........362
Horton, David B............ 62
Howell, Joseph L............ 62
Kellogg, Charles M..........700
Krug, J. F...................428
Lapponi, Guiseppe...........362
LaWall, Elbert W...........559
MacDonald, Alexander E....362
McCaw, James B.............117
Meisburger, William C. L....361
Myers, William H............507
Osiris, Daniel................507
Otis, William K..............239
Packwood, William Jasper....239
Peltier, Pierre Desnoyers.....117
Potter, Emily Bostwick......294
Robbins, Alfred H...........362
Rodman, Harry Heath.......298
Tompkins, Orrin A. .........760
Thomas, William B..........559
Wiggins, John H............632
Woolsey, Elliott H..........429
Wyeth, John ................700

Occipito-posterior positions, new management of ...............421
Operation, consent to............620
Otis, William Kelly, in memoriam.358
Otitis media, acute ..............344
aspiration in acute..624
Otorrhea, how to manage........547
Ovarian grafting, heteroplastic...393
Ovariotomy, double .............417
Oxidation, systemic promotion of.110

PANCREATIC CYST, case of..665
Park, Roswell, story of discovery of circulation............ 20
Patrick case, the................623

**Personal:**

Bowerman, Edward A.; Griffith, A. C.; Armour, Watson; Wilson, Nelson W.; Pohlman, Julius................ 62
Brown, John Young; Haase, Charles; Nottingham, William; Walker, J. E.; Park, R.506

PAGE

Chalmers, John; MacLeod, James A.; Twohey, John J.; Follonsbee, F.; Watson, William H.; Senn, Nicholas; House, William; Dudley, E. C. ....................699
Doty, Alvah H.; Fairbairn, John Fitzgerald; Keen, W. W ....................427
Evans, William A.; Ellis, Charles A.; Cooke, Almon H.; McKee, E. S. ..........630
Foster, Carlton; Danser, Earl G.; Wells, Brooks H.; Potter, Irving W.; Ely, C. S.; O'Brien, John D.; Venezia, Professor di ..............361
Hess, L. T.; Marchiavafa, Ettore; Brown, John Young; Carpenter, Archibald D.; Goldberg, S.; Clements, Charles A.; Stockton, Charles G. ..............428
House, William..............175
Howard, Charles F.; O'Donnell, W. J.; Pohlman, Augustus Grote ..............757
Lewis, James H.; Wende, Ernest; Fronczak, Francis E.; Goldberg, Jacob; Gaertner, William; Card, D. P...297
McKelway, St. Clair.........115
McMurtry, L. S.; Pryor, John H.; Bremerman, L. W.; Meinesbach, R. O.........296
McMurtry, L. S.; Reed, Charles A. L.; Hanley, L. G.; Lewis, F. Park; Ferguson, A. H.; Wilson, Nelson W.; Wende. Ernest....174
Pillsbury, C. W.; Sharp, H. P.; Smith, Eugene A.; Lewis, F. Park; Wende, H. S.; Williams, Herbert U.; Schwarz, Henry; Dorsett, Walter B.; Torrey, Edward; Foster, William S..........116
Putnam, James Wright; Bender, Ida C.; Walker, Edwin; Dorsett, Walter Blackburn; Bourne, C. W.; Bryant, Joseph Decatur; Bainbridge, William Seaman ..........756
Smith, Eugene A.; Card, N. P.360
Thompson, J. Ford..........295
Townsend, Wisner R.; Fitch, W. E.; Park, Roswell; O'Reilly, Robert M.; Gaylord, H. R.; Straub, Paul F.238
Vander Veer, Albert; Grove, Benjamin H.; Grant, H. Y..699

PAGE

White, J. H.; Bowman, Carlos E., Dike, I. A. M.; Kenerson, Vertner; LeBreton, Prescott ................557
Woodbury, John McGaw.....237

Phosphorus, organic, in nervous diseases ......................549
Phthisis night sweats, treatment of ..........................736
Physician-civilian ...............723
Physicians' services, various values of.....................622
Pilcher, James Evelyn, training of medical officer of state forces.191
Porter, Miles F., concernig amputations ........................730
Pozzi "Live d'Or" ..............294
Pregnancy after operation for gonorrheal salpingitis .274
 cornual ..............274
 death during ........416
 diagnosis of early.....617
Privileged communication, waiver of by heir....................619

**Progress in Medical Science:**
 Orthopedic Surgery, by Prescott LeBreton.............. 91
 Pediatrics, by Maud J. Frye..154
 Obstetrics and Gynecology, by E. S. McKee .......274, 416
 Forensic Medicine, by E. S. McKee ..............618, 679
Prostatic hypertrophy treatment. .87
Pruritus ani ....................354
Puerperal fever, intravenous injections of collargolum in ....................224
 sepsis, surgical and serum treatment of.....473
Putnam, James Wright, etiology of functional nervous diseases.. 71
Pyrenol, influenza treated with...737
 in influenza..............623
 therapeutics of.......... 95
Pylorus, obstruction in region of.465

QUACKERY, bill to stop.......621

RAILWAY STATION, Buffalo's a disgrace ...................555
Rogers, Dr. Howard J., letter relating to reciprocity in medical licensure ....................163
Regents' academic examinations..281
Reciprocity in medical licensure ........................315, 482
Reminiscences, some professional.533
Report of state commissioner of health ........................505

PAGE
Respiratory tract, diseases of.....345
Rooker, A. M., fracture of skull..403
Roosa, D. B. St. John, monocular hysterical amblyopia ...........673
Roosevelt, President inspects the canal zone ....................349
Rose-Cook, cerebral abscess .....389

SANDER (Enno) prize essay, 1906 .........................191
San Francisco physicians, needs of ............................ 61
Sciatica, treatment of ............733
Scurvy, infantile ................377
Sewage, danger of...............555
Serum, therapeutics of artificial...161
antitetanic for lockjaw....348
Shoemaker, John V., carcinoma cutis ..........................672
Shrady, George F., some professional reminiscences............533
Shurly, B. R., anemia and incipient tuberculosis, treatment by hypodermic medication..............537
Silver therapy ...................277
Skull, fracture of with complications ..........................403
Smith, Eugene A., surgical aspect of gastric ulqer...............445
Social evil ......................249

**Society Meetings:**

Allegany Society of Buffalo..432
American Academy of Medicine .....................635
American Association of Obstetricians and Gynecologists ..................63, 761
American Climatological Association ..................635
American Congress of Physicians and Surgeons......563
American Medical Association ......................563, 700
American Medical Editors' Association ...............634
American Roentgen Ray Society .................. 65
Association of American Medical Colleges ..............634
Association of Military Surgeons, U. S................117
Buffalo Academy of Medicine 64, 175, 239, 299, 363, 431, ..........508, 562, 634, 701, 760
British Medical Association .......................64, 118
Buffalo Nurses' Association..559
Eleventh Antialcoholic Congress .....................761
Esculapian Club ............432

PAGE
Harvard Medical Society of New York.................432
Homeopathic Medical Society State of New York.........239
International Homeopathic Congress ..................118
Lake Keuka Medical Association ...................... 65
Lehigh Valley Railway Surgeons Association..........299
Medical Association of Central New York............175
Medical Society County of Erie ..........176, 364, 634, 760
Medical Society County of Niagara ...............65, 298
Medical Society County of Steuben ...................701
Medical Society County of Wyoming .................300
Medical Society State of New York .............298, 363, 429
Medical Society State of N. Y., eighth district branch...118
Medical Union of Buffalo....118
Mississippi Valley Medical Association ...........118, 299
National Association of U. S. Pension Examining Surgeons .....................563
National Confederation State Medical Examining and Licensing Boards ..........635
Niagara Falls Academy of Medicine ..................701
Philippine Islands Medical Association ...............563
Rochester Academy of Medicine ......................700
Roswell Park Medical Club..239
Sanitary Officers of the State of N. Y...................175
Sixth International Dermatological Congress ....563, 761
Society of Clinical Surgery...632
Southern Surgical and Gynecological Association ..298, 363
Western N. Y. Homeopathic Medical Society...........634
Women's Medical Society State of N. Y..............562

**Society Proceedings:**

Medical Association of Central New York ................739
Medical Society County of Erie ..........52, 404, 561, 675

Soothing syrups, successful.......619
Spelling, simplified, and the medical profession.................326

PAGE
Spitting on sidewalks, ordinance against ....................... 556
Spleen, puerperal strangulation of. 418
State Board of Medical Examiners ............................ 755
Stover, Charles, relation of tuberculosis to municipal and industrial life ......................... 583
Stoves, pipeless ................. 556
Streets, use of as playgrounds
Student ideals .................. 183
prohibited ..................... 359
Surgical instruments ............ 402

TEXAS board of medical examiners ......................... 697
Thoma, Fridolin, leading men and events in history of American medicine ................. 40
Tonsil, acute infections of pharyngeal ......................... 158
Toronto university confers degrees ......................... 295
Totman, D. M., expert medical testimony ..................... 321
Tooth decay, prevention of ...... 388
Tuberculosis, dust as a cause of.... 497
relation of to municipal and industrial life ................ 583
management of pulmonary ........... 135

**Topic of Public Interest:**

Blindness, prevention of unnecessary ............. 491, 493
Delegates, House of and Medical Society County of Erie. 489
Free university, a ............ 279
Medical licensure, various views in ... ............... 482
Quack doctors, safeguards to protect people from........ 98
Reciprocity in medical licensure .................. 162, 483
The Isthmian canal .......... 349
What to eat ................ 688

UROTROPIN and helmitol..... 278

VANDER VEER, Albert, reciprocity in medical licensure.. 315
Version or high forceps.......... 529
Visions of Mary Czajka......... 470
Vital statistics, state file of...... 505
Vulva, pilocarpine in pruritus of.. 692

WALLACE, W. L., intestinal resection and anastomosis, rapid and safe method.......... 517
Walsh, John J., in memorian..... 54
Warbrick, John C., treatment of chronic infections of nasopharynx ....................... 80
Water, pure drinking............ 359
Whooping cough, leukocytosis in. 154
Why not? A plea.............. 335
Wilder, Burt G., medical profession and simplified spelling..... 326
Witness, suggestions to medical.. 685
Woodbury, Frank Thomas, Cesarean operation under difficulties. 726
Wounds, dressing of............ 421
Wright, Adam H., accidental obstetric hemorrhage............ 257

YOUNG, J. C., management of pulmonary tuberculosis ...... 135

